# Urinary HSP90 as a non-invasive biomarker of active tubular injury in kidney transplant recipients: A multicenter prospective study

**DOI:** 10.1016/j.cstres.2026.100209

**Published:** 2026-08-26

**Authors:** Kimihito Tachikawa, Toshiaki Tanaka, Takeshi Maehana, Tetsuya Shindo, Yuki Kyoda, Kohei Hashimoto, Hayato Nishida, Hiroshi Kitamura, Takahiro Tsuji, Naoya Masumori

**Affiliations:** 1 Department of Urology, Sapporo Medical University School of Medicine, Sapporo, Japan; 2 Department of Urology, National Hospital Organization Hokkaido Medical Center, Sapporo, Japan; 3 Department of Urology, Yamagata University Faculty of Medicine, Yamagata, Japan; 4 Department of Urology, University of Toyama, Toyama, Japan; 5 Department of Pathology, Sapporo City General Hospital, Sapporo, Japan

**Keywords:** Kidney transplantation, HSP90, Urine biomarker, Tubular injury, Allograft pathology, Banff classification

## Abstract

We previously demonstrated that serum heat shock protein 90 (HSP90) is a promising biomarker for severe acute allograft rejection involving vascular endothelial injury; however, the clinical significance of urinary HSP90 (uHSP90) remains unclear. We hypothesized that uHSP90 reflects acute T cell-mediated rejection (ATCMR), a common form of allograft rejection characterized by tubulitis. This multicenter prospective study included 262 paired urine and serum samples from 112 kidney transplant recipients, along with corresponding renal allograft biopsy specimens. uHSP90 levels were measured and normalized to urinary creatinine (uHSP90/Cr). Histopathological findings were evaluated according to the Banff (2022) classification, and HSP90 expression was assessed by immunohistochemistry. uHSP90/Cr levels were significantly elevated in conditions associated with active tubular injury, including ATCMR, calcineurin inhibitor nephropathy, and BK virus nephropathy, although they were not specific to ATCMR. uHSP90/Cr levels were significantly associated with Banff scores for interstitial inflammation, tubulitis, interstitial fibrosis, and tubular atrophy. In contrast, renal tissue HSP90 expression was significantly decreased and negatively correlated with uHSP90/Cr levels (ρ = −0.29, *P* < .001). uHSP90/Cr demonstrated moderate diagnostic performance for active tubular injury, with an area under the receiver operating characteristic curve of 0.713, comparable to that of β2-microglobulin and superior to that of N-acetyl-β-D-glucosaminidase. Multivariable analysis demonstrated that detectable uHSP90 was independently associated with active tubular injury (odds ratio, 4.93; 95% confidence interval, 2.21–11.00; *P* < .001). These findings suggest that uHSP90 may serve as a biomarker of renal allograft tubular injury, although it is not specific for allograft rejection.

## Highlights

• Urinary HSP90 is a novel biomarker of active tubular injury in kidney allografts.
• Urinary HSP90 correlates with Banff inflammation and tubular injury scores.
• Tissue HSP90 expression inversely correlated with urinary HSP90 levels.
• Detectable urinary HSP90 is independently associated with active tubular injury.
• Urinary HSP90 performs comparably with established tubular injury biomarkers.

## Background

Kidney transplantation is the optimal treatment for patients with end-stage renal disease. However, long-term graft survival remains limited by various forms of allograft injury, including rejection, drug toxicity, and infection.^1^, ^2^, ^3^ Early detection of graft injury is critical for timely therapeutic intervention and improved outcomes. Currently, allograft biopsy remains the gold standard for diagnosing the underlying causes of graft dysfunction.^4^ However, biopsy is invasive, carries a risk of complications, and is unsuitable for frequent monitoring. Therefore, there is a strong clinical need for reliable non-invasive biomarkers that can reflect ongoing graft injury and support clinical decision-making. Although serum creatinine (sCr) is widely used to assess graft function, it is a non-specific and relatively late marker that does not directly reflect underlying pathological changes. Moreover, sCr cannot reliably distinguish among different causes of graft dysfunction, such as rejection, calcineurin inhibitor toxicity, and viral nephropathy.

Heat shock protein 90 (HSP90) is a molecular chaperone involved in the stabilization and activation of numerous client proteins and plays a critical role in cellular stress responses.^5^ Recent evidence has identified HSP90 as a central regulator of proteostasis, coordinating protein folding, stabilization, and degradation in response to cellular stress. Through these functions, HSP90 contributes to the maintenance of cellular homeostasis and adaptation to tissue injury.^6^ We previously reported that serum HSP90 (sHSP90) levels are specifically elevated in acute rejection after kidney transplantation, particularly in cases involving vascular injury, suggesting that alloreactive injury targeting vascular endothelial cells promotes the release of HSP90 into the circulation.^7^ However, sHSP90 appears to be less sensitive to tubulointerstitial injury, a hallmark of acute T cell-mediated rejection (ATCMR) and several other forms of graft injury. As tubular epithelial cells are directly exposed to the urinary space, we hypothesized that tubular injury may lead to the release of HSP90 into the urine, resulting in increased urinary HSP90 (uHSP90) levels. Accordingly, uHSP90 may serve as a non-invasive biomarker of tubular injury in kidney allografts.

The aim of this study was to evaluate uHSP90 levels in kidney transplant recipients and assess their utility as a non-invasive biomarker of renal allograft injury by comparing them with histopathological findings, including Banff classification scores and HSP90 immunohistochemical expression.

## Results

### Patient characteristics

A total of 262 paired urine and serum samples with corresponding renal allograft biopsy specimens from 112 kidney transplant recipients were included in this study. Baseline characteristics were analyzed for 255 samples with complete clinical and laboratory data (Table 1).

**Table 1:** Demographic and clinical characteristics of the study population.

Variable			Total (*N* = 255)
*Demographics*			
	Age at biopsy, y		52 [43-61]
	Sex (male/female), n (%)		163 (64)/92 (36)
	Time from transplantation to biopsy, d		188 [31-373]
*Laboratory data*			
	Serum creatinine, mg/dL		1.39 [1.09-1.67]
	Urinary creatinine, mg/dL		71.7 [39.6-108.5]
	Urinary HSP90, ng/mL		0 [0.000-0.114]
	Urinary HSP90/Cr, pg/mg creatinine		0 [0.000-0.202]
	Serum HSP90, ng/mL		36.9 [25.8-54.1]
	IHC positivity, %		10.2 [1.15-27.4]
*Biopsy type, n (%)*			
	Protocol biopsy		200 (78)
	Indication biopsy		55 (22)
*Pathological findings, n (%)*			
	Acute rejection		16 (6.3)
		T cell-mediated rejection	12 (4.7)
		Antibody-mediated rejection	4 (1.6)
	Calcineurin inhibitor nephropathy		25 (9.8)
	BK virus nephropathy		5 (2.0)
	IF/TA		52 (20.4)
	Borderline change		14 (5.5)

Data are presented as median [interquartile range] or number (percentage). Urinary HSP90/Cr was calculated by normalizing urinary HSP90 to urinary creatinine and is expressed as pg/mg creatinine (values in ng/mg creatinine multiplied by 1000 for readability). IHC positivity was available for 208 samples, serum creatinine was available for 253 samples, and all other variables were available for 255 samples. HSP90, heat shock protein 90; Cr, creatinine; IHC, immunohistochemistry; IF/TA, interstitial fibrosis and tubular atrophy.

### uHSP90 levels across pathological diagnoses

**Fig. 1:**
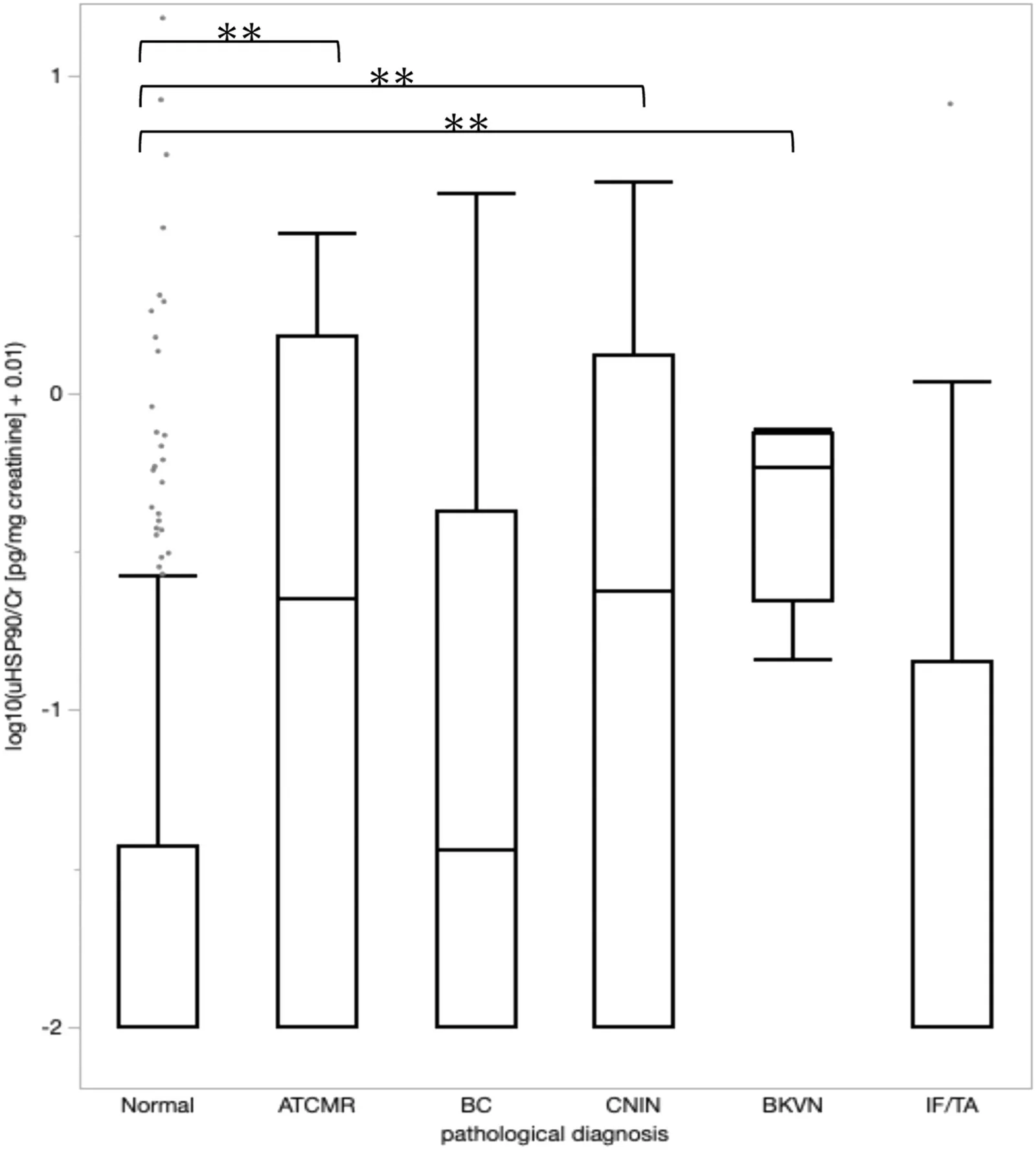
Creatinine-normalized urinary HSP90 levels across pathological diagnoses. Urinary HSP90 was measured by enzyme-linked immunosorbent assay and normalized to urinary creatinine, expressed as pg/mg creatinine. For visualization, values are shown as log10(uHSP90/Cr + 0.01). Box plots indicate the median and interquartile range (IQR), with whiskers representing 1.5 × IQR; individual data points are overlaid. Comparisons between the normal group and each pathological group were performed using the Mann–Whitney *U* test. *P < .05 and **P < .01 vs. the normal group. uHSP90, urinary heat shock protein 90; Cr, creatinine; ATCMR, acute T-cell-mediated rejection; BC, borderline change; BKVN, BK virus nephropathy; CNIN, calcineurin inhibitor nephropathy; IF/TA, interstitial fibrosis and tubular atrophy.

uHSP90/Cr levels differed significantly among the pathological diagnostic categories (Figure 1, Table 2). Compared with the normal group, uHSP90/Cr levels were significantly elevated in patients with ATCMR. Furthermore, uHSP90/Cr levels were also significantly increased in patients with calcineurin inhibitor nephropathy (CNIN) and BK virus nephropathy (BKVN) (Mann–Whitney *U* test, *P < .05, **P < .01). In contrast, no significant differences were observed in the borderline change (BC) or interstitial fibrosis and tubular atrophy (IF/TA) groups.

**Table 2:** Comparison of HSP90 levels and clinical parameters by pathological diagnosis.

Variable	Normal (*n* = 169)	ATCMR (*n* = 12)	BC (*n* = 14)	ABMR (*n* = 4)	CABMR (*n* = 4)	CNIN (*n* = 21)	BKVN (*n* = 5)	IF/TA (*n* = 40)
Urinary HSP90, ng/mL	0.00 [0.00-0.07]	0.13 [0.00-0.43]**	0.04 [0.00-0.21]	0.08 [0.02-0.19]	0.00 [0.00-0.07]	0.10 [0.00-0.53]**	0.41 [0.17-0.60]**	0.00 [0.00-0.06]
Urinary creatinine, mg/dL	78.7 [45.3-121.6]	38.5 [20.8-86.9]*	74.0 [26.9-144.1]	59.8 [12.5-109.5]	79.0 [44.6-106.3]	44.9 [29.4-84.0]*	71.7 [69.8-82.4]	62.9 [40.8-100.2]
Urinary HSP90/Cr, pg/mg creatinine	0.00 [0.00-0.103]	0.22 [0.00-1.50]**	0.06 [0.00-0.46]	0.40 [0.02-1.36]*	0.00 [0.00-0.12]	0.00 [0.00-1.30]**	0.57 [0.23-0.74]**	0.00 [0.00-0.13]
Detectable uHSP90, *n* (%)	40 (25.8)	8 (66.7)**	7 (50.0)	3 (75.0)	1 (25.0)	12 (57.1)**	5 (100)**	11 (27.5)
Serum HSP90, ng/mL	42.5 [28.0-59.7]	31.2 [20.2-52.9]	42.8 [24.6-59.5]	26.5 [15.4-32.1]*	27.0 [14.6-48.7]	22.7 [13.0-33.2]**	31.0 [18.4-51.0]	37.7 [25.6-48.5]
IHC positivity, %	15.3 [3.1-30.4]	0.34 [0.1-5.3]**	23.1 [2.0-31.0]	1.97 [0.94-23.4]	29.4 [5.8-46.9]	0.40 [0.12-2.74]**	0.93 [0.06-16.0]*	10.8 [0.60-34.8]
Serum creatinine, mg/dL	1.36 [1.06-1.58]	2.75 [1.75-5.03]**	1.45 [1.30-1.68]	1.06 [0.93-2.32]	1.68 [1.50-2.04]*	1.25 [0.97-1.80]	2.13 [1.5-2.24]*	1.41 [1.10-1.70]

Data are presented as median [interquartile range] or *n* (%). Differences between the normal group and each pathological group were analyzed using the Mann-Whitney *U* test for continuous variables and Fisher’s exact test for detectable uHSP90. The normal group included samples without acute or chronic rejection, CNIN, BKVN, IF/TA, or BC. Urinary HSP90/Cr was calculated by normalizing urinary HSP90 to urinary creatinine and is expressed as pg/mg creatinine (values in ng/mg creatinine were multiplied by 1000 for readability). Detectable uHSP90 was defined as urinary HSP90 > 0 ng/mL. *P < .05 vs. the normal group; **P < .01 vs. the normal group. IHC data were available for 208 samples (normal, *n* = 117; ATCMR, *n* = 10; BC, *n* = 11; ABMR, *n* = 4; CABMR, *n* = 4; CNIN, *n* = 24; BKVN, *n* = 5; IF/TA, *n* = 33). HSP90, heat shock protein 90; IHC, immunohistochemistry; ATCMR, acute T-cell-mediated rejection; ABMR, acute antibody-mediated rejection; CABMR, chronic active antibody-mediated rejection; CNIN, calcineurin inhibitor nephropathy; BKVN, BK virus nephropathy; IF/TA, interstitial fibrosis and tubular atrophy; BC, borderline change.

### Immunohistochemical expression of HSP90

**Fig. 2:**
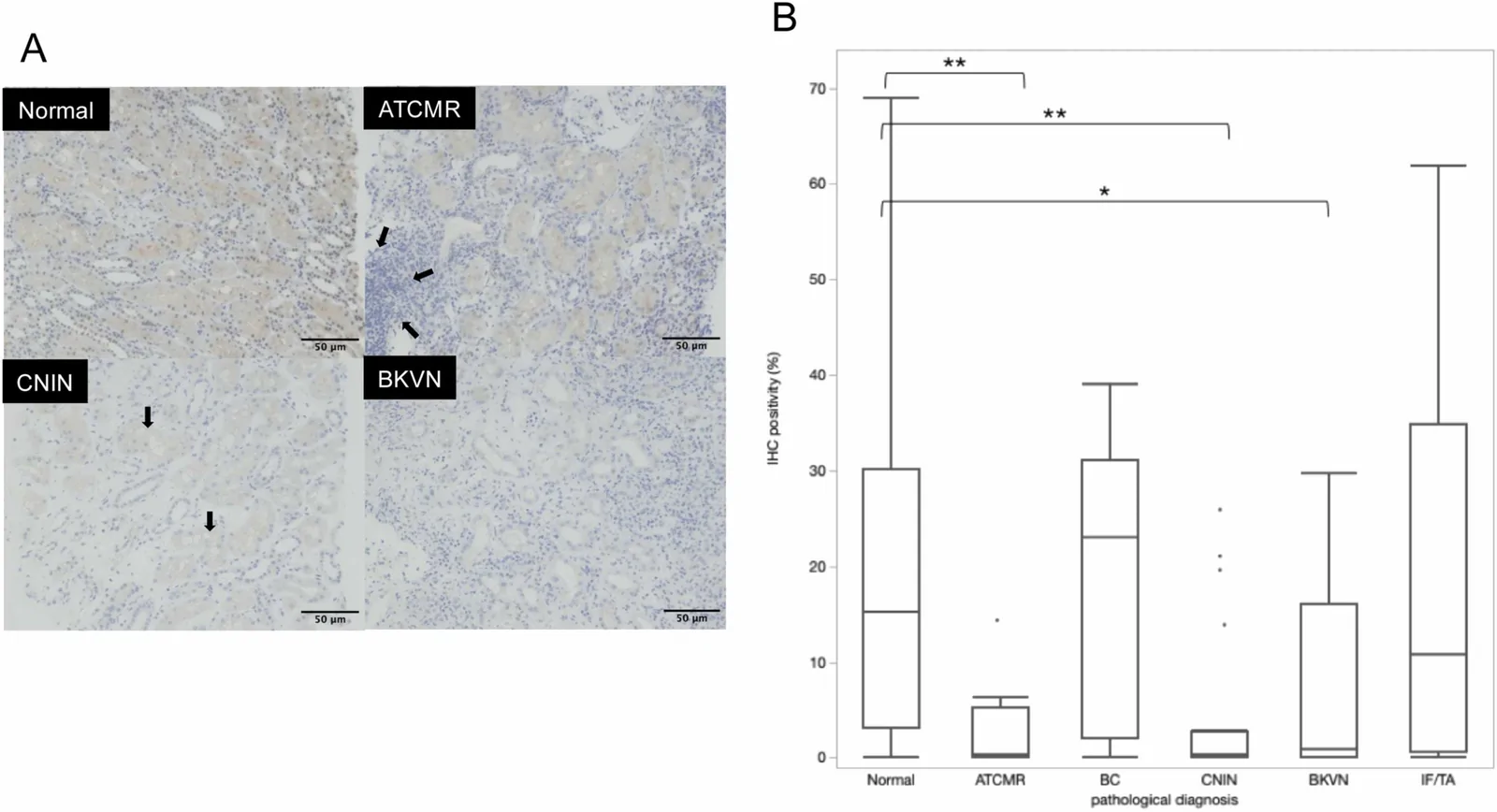
Immunohistochemical expression of HSP90 in renal allograft tissue. (A) Representative HSP90 immunohistochemistry images from renal allograft biopsies with normal histology, acute T cell-mediated rejection (ATCMR), calcineurin inhibitor nephropathy (CNIN), and BK virus nephropathy (BKVN). HSP90 immunoreactivity was observed predominantly in tubular epithelial cells. Arrows in the ATCMR panel indicate areas of dense inflammatory cell infiltration without discernible HSP90 immunoreactivity, whereas arrows in the CNIN panel indicate tubular epithelial vacuolization. Scale bars, 50 μm. (B) The percentage of HSP90-positive area in renal biopsy specimens was quantified by immunohistochemistry (IHC). Box plots indicate the median and interquartile range (IQR), with whiskers representing 1.5 × IQR; individual data points are overlaid. HSP90 positivity is significantly lower in the ATCMR, CNIN, and BKVN groups than in the normal group (Mann–Whitney *U* test). *P < .05 and **P < .01 vs. the normal group. IHC, immunohistochemistry; ATCMR, acute T-cell-mediated rejection; BC, borderline change; CNIN, calcineurin inhibitor nephropathy; BKVN, BK virus nephropathy; IF/TA, interstitial fibrosis and tubular atrophy.

Representative HSP90 immunohistochemistry images from renal allograft biopsies with normal histology, ATCMR, CNIN, and BKVN are shown in Figure 2A. HSP90 immunoreactivity was observed predominantly in tubular epithelial cells, with no clearly discernible differences among tubular segments. No discernible HSP90 staining was observed in infiltrating inflammatory cells. The percentage of HSP90-positive area differed among the diagnostic groups (Figure 2B). HSP90 positivity was significantly lower in the ATCMR, CNIN, and BKVN groups than in the normal group (Mann–Whitney *U* test, *P < .05, **P < .01).

### Correlation analyses of uHSP90

**Fig. 3:**
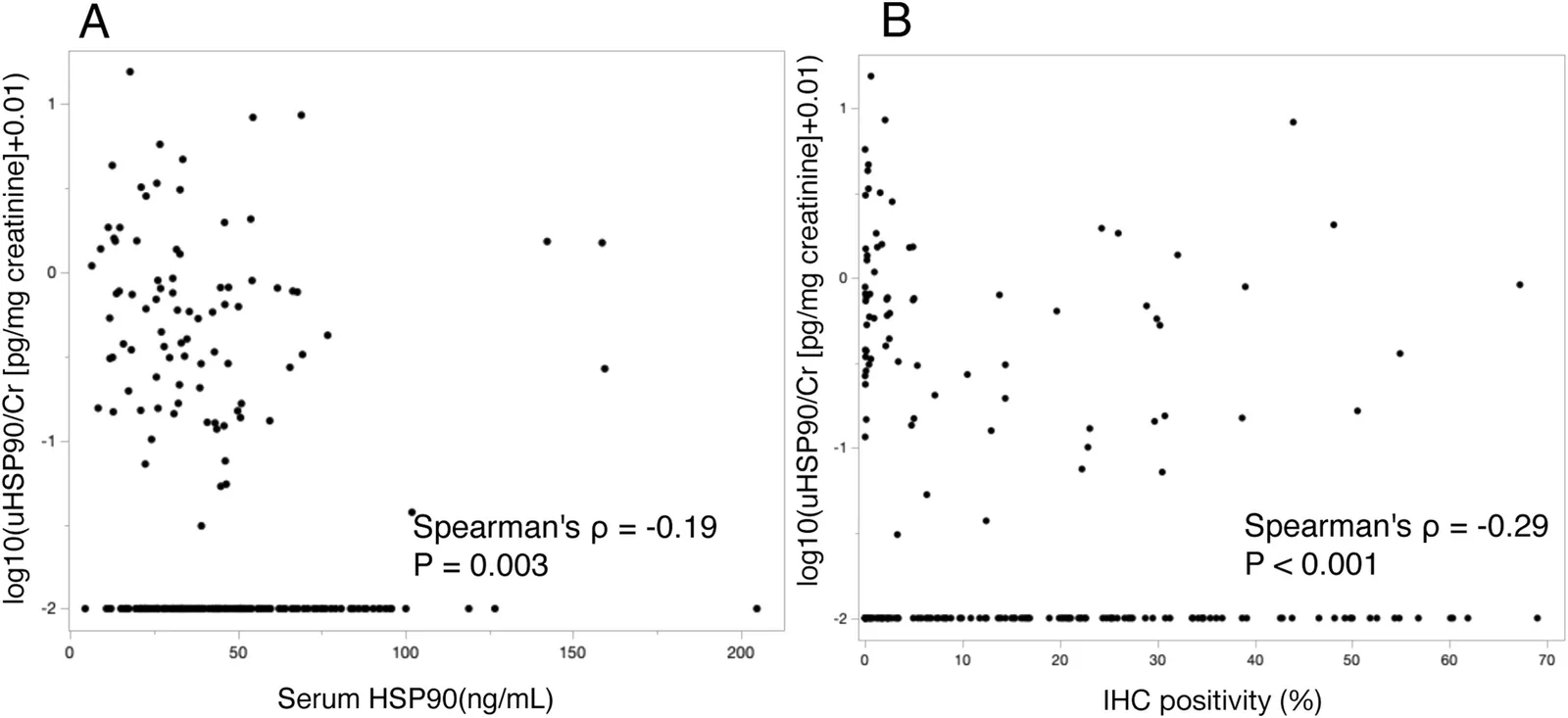
Correlation analyses of creatinine-normalized urinary HSP90. Urinary HSP90 was normalized to urinary creatinine and expressed as pg/mg creatinine. For visualization, values are shown as log10(uHSP90/Cr + 0.01). Scatter plots show the correlations between log10(uHSP90/Cr + 0.01) and (A) serum HSP90 (ng/mL) and (B) HSP90 immunohistochemical positivity (%). Spearman’s rank correlation coefficient (ρ) and *P* values are shown in each panel. uHSP90, urinary heat shock protein 90; Cr, creatinine; IHC, immunohistochemistry; HSP90, heat shock protein 90.

Correlation analyses were performed between log-transformed uHSP90/Cr levels and other parameters (Figure 3). uHSP90/Cr levels showed a weak negative correlation with sHSP90 levels (Spearman’s ρ = −0.19, *P* = .003). A stronger negative correlation was observed between uHSP90/Cr levels and HSP90 immunohistochemical positivity in renal tissue (Spearman’s ρ = −0.29, *P* < .001).

### Association between uHSP90 and Banff scores

**Fig. 4:**
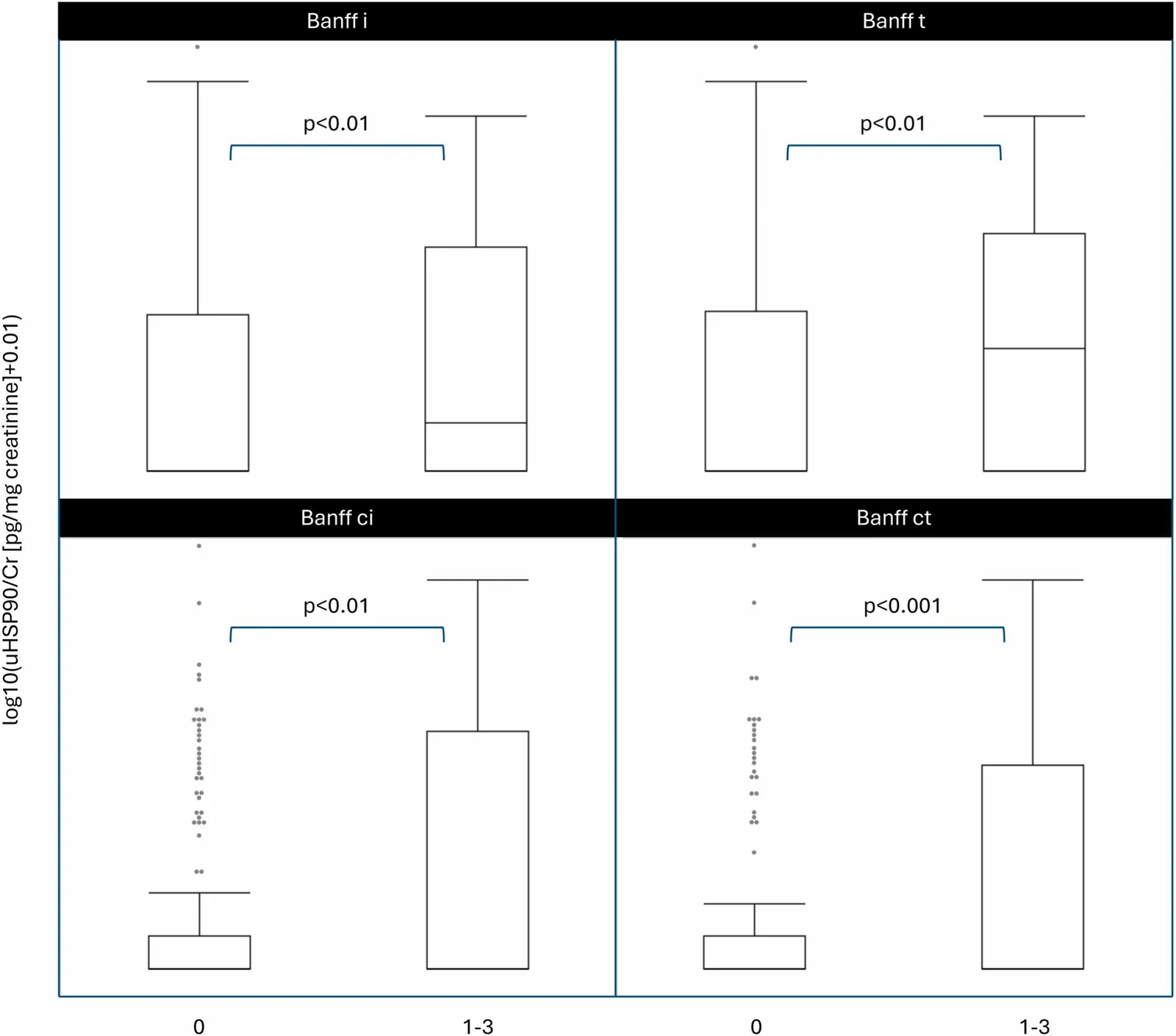
Creatinine-normalized urinary HSP90 levels according to Banff scores. Urinary HSP90 was measured by enzyme-linked immunosorbent assay and normalized to urinary creatinine, expressed as pg/mg creatinine. For visualization, values are shown as log10(uHSP90/Cr + 0.01). Box plots indicate the median and interquartile range (IQR), with whiskers representing 1.5 × IQR; individual data points are overlaid. Urinary HSP90/Cr levels were compared between specimens with a Banff score of 0 and those with scores of 1–3 for each lesion (i, t, ci, and ct) using the Mann–Whitney *U* test. *P* values are shown in each panel. uHSP90, urinary heat shock protein 90; Cr, creatinine; Banff i, interstitial inflammation; Banff t, tubulitis; Banff ci, interstitial fibrosis; Banff ct, tubular atrophy.

uHSP90/Cr levels were significantly associated with histopathological Banff scores (Figure 4). uHSP90/Cr levels were significantly higher in patients with positive scores (1–3) than in those with a score of 0 for interstitial inflammation (i, *P* < .01), tubulitis (t, *P* < 0.01), interstitial fibrosis (ci, *P* < .01), and tubular atrophy (ct, *P* < .001).

### Analyses according to biopsy type

Among the 255 biopsies, 200 were protocol biopsies and 55 were indication biopsies. Active tubular injury was more frequently observed in indication biopsies than in protocol biopsies (18/55 [32.7%] vs. 21/200 [10.5%], respectively; Fisher’s exact test, *P* < .001). Among protocol biopsies, uHSP90/Cr was significantly higher in biopsies with active tubular injury than in those without active tubular injury (median [IQR], 0.519 [0–1.008] vs. 0 [0–0.116] pg/mg creatinine; *P* < .001). Detectable uHSP90 was also more frequent in biopsies with active tubular injury (14/21 [66.7%] vs. 52/179 [29.1%]; *P* = .001). In contrast, sCr did not differ significantly according to the active tubular injury status (1.38 [1.02–1.73] vs. 1.30 [1.04–1.51] mg/dL; *P* = .486). Among indication biopsies, uHSP90/Cr (0.299 [0–1.481] vs. 0 [0–0.078] pg/mg creatinine; *P* = .004), the frequency of detectable uHSP90 (12/18 [66.7%] vs. 9/37 [24.3%]; *P* = .004), and sCr (2.69 [1.75–4.42] vs. 1.70 [1.46–2.33] mg/dL; *P* = .017) were all significantly higher in biopsies with active tubular injury than in those without active tubular injury (Supplementary Table 1). sCr data were unavailable for two indication biopsies with active tubular injury.

### Individual-level changes in repeated biopsies

Among the 112 patients, 87 underwent more than 1 biopsy. Nineteen patients had 24 consecutive biopsy pairs in which the active tubular injury status changed. The direction of change in uHSP90/Cr was concordant with the change in the active tubular injury status in 11 pairs (45.8%), discordant in 11 pairs (45.8%), and unchanged in 2 pairs (8.3%). Thus, changes in uHSP90/Cr did not consistently correspond to changes in the active tubular injury status at the individual-patient level.

### Multivariable analysis of factors associated with active tubular injury

Multivariable logistic regression identified independent factors associated with active tubular injury, defined as ATCMR, CNIN, or BKVN (Table 3). Detectable uHSP90 (> 0 ng/mL) was independently associated with active tubular injury (odds ratio [OR], 4.93; 95% confidence interval [CI], 2.21–11.00; *P* < .001). sCr was also independently associated with active tubular injury (OR, 2.28; 95% CI, 1.23–4.25; *P* = .009). In contrast, age at biopsy (adjusted OR per year, 1.00; 95% CI, 0.97–1.03; *P* = .941), time from transplantation to biopsy (adjusted OR per year, 0.64; 95% CI, 0.38–1.08; *P* = .095), and biopsy type (indication vs. protocol biopsy: adjusted OR, 2.10; 95% CI, 0.77–5.75; *P* = .150) were not significantly associated with active tubular injury.

**Table 3:** Multivariable logistic regression predicting active tubular injury.

Variable	OR (95% CI)	*P* value
Detectable uHSP90 (> 0 ng/mL)	4.93 (2.21-11.00)	<.001
Serum creatinine, mg/dL	2.28 (1.23-4.25)	.009
Age at biopsy, y	1.00 (0.97-1.03)	.94
Time from transplantation to biopsy, y	0.64 (0.38-1.08)	.095
Biopsy type, indication vs. protocol	2.10 (0.77-5.75)	.15

Multivariable logistic regression analysis was performed for all cases (*n* = 253). Detectable uHSP90 was defined as urinary HSP90 > 0 ng/mL. Active tubular injury was defined as acute T-cell-mediated rejection, calcineurin inhibitor nephropathy, or BK virus nephropathy. The time from transplantation to biopsy was rescaled in years for the regression analyses. Biopsy type was categorized as protocol or indication biopsy, with protocol biopsy as the reference category. Adjusted ORs with 95% confidence intervals are shown. uHSP90, urinary heat shock protein 90; OR, odds ratio; CI, confidence interval.

### Diagnostic performance of uHSP90 for active tubular injury in kidney allografts

**Fig. 5:**
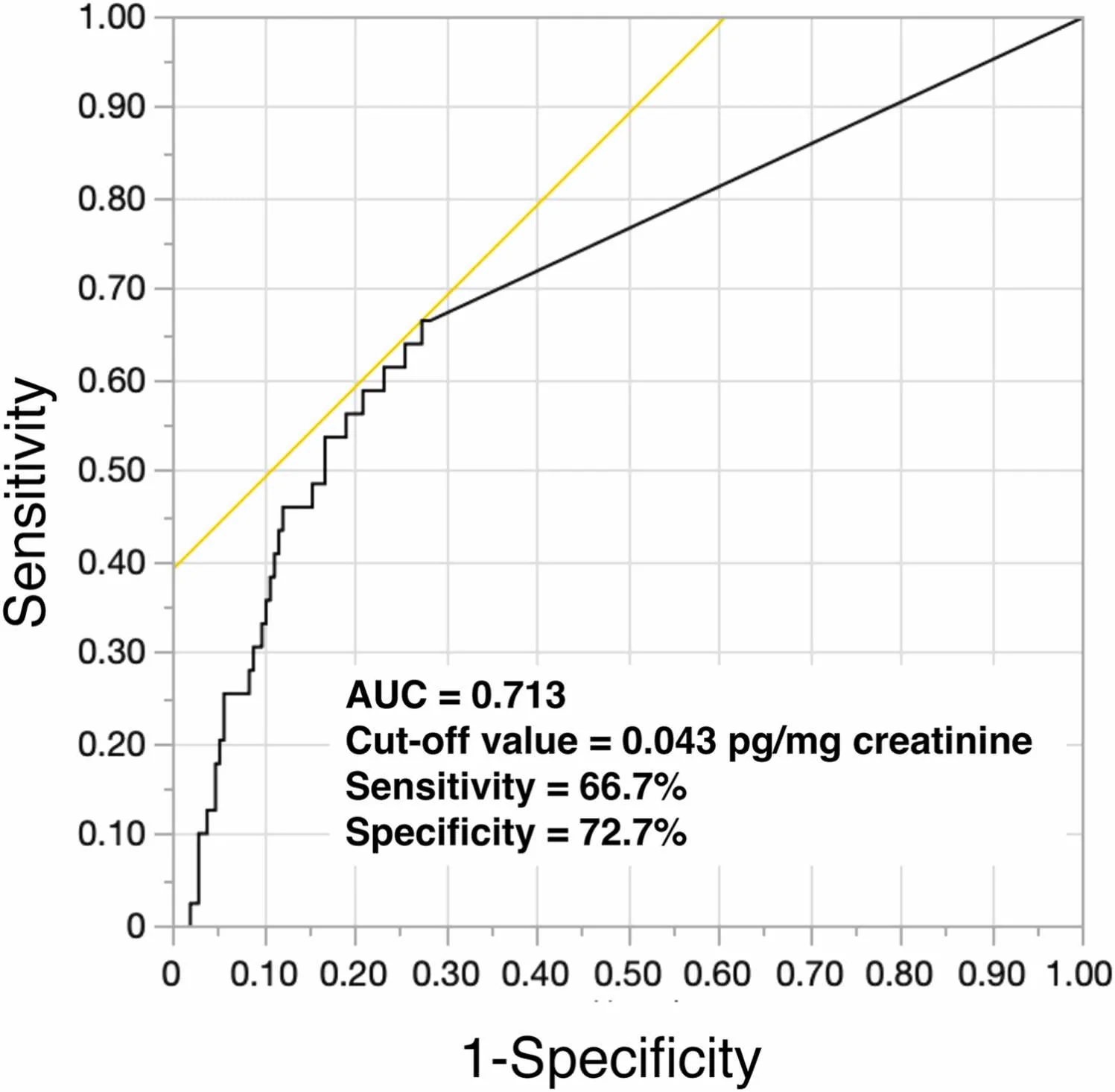
Receiver operating characteristic (ROC) curve of creatinine-normalized urinary HSP90 for detecting active tubular injury. The ROC curve shows the ability of urinary HSP90/Cr to discriminate active tubular injury (acute T-cell-mediated rejection, calcineurin inhibitor nephropathy, or BK virus nephropathy) from all other pathological diagnoses. The area under the curve is 0.713. At the optimal cutoff value of 0.043 pg/mg creatinine, the sensitivity and specificity are 66.7% and 72.7%, respectively. AUC, area under the curve.

**Fig. 6:**
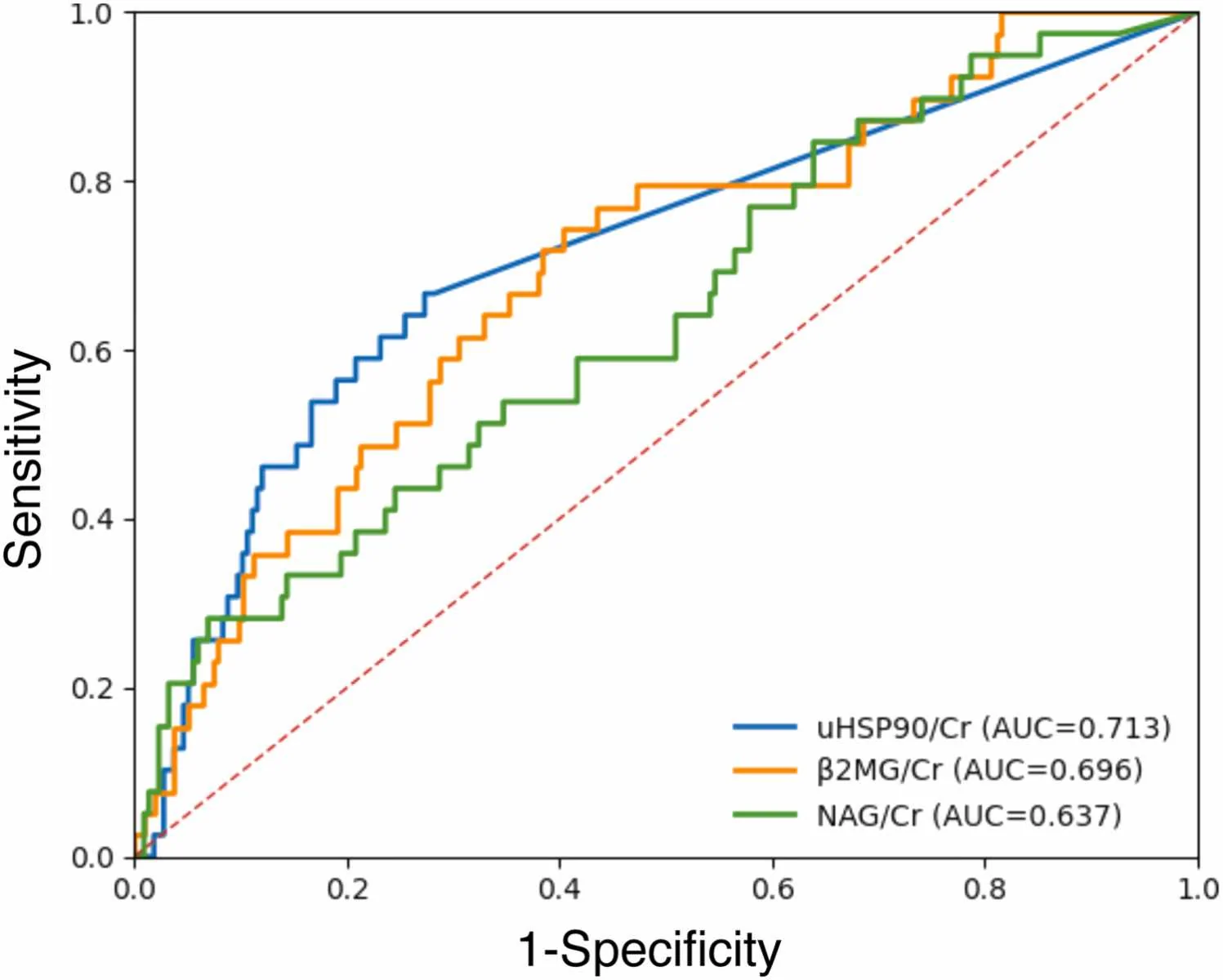
Receiver operating characteristic (ROC) curves comparing creatinine-normalized urinary biomarkers for detecting active tubular injury. ROC curves were generated to evaluate the ability of creatinine-normalized urinary biomarkers to discriminate active tubular injury (acute T-cell-mediated rejection, calcineurin inhibitor nephropathy, or BK virus nephropathy) from all other pathological diagnoses. ROC curves are shown for uHSP90/Cr (pg/mg creatinine), β2MG/Cr (μg/mg creatinine), and NAG/Cr (IU/mg creatinine). The areas under the curve (AUCs) are 0.713, 0.696, and 0.637 for uHSP90/Cr, β2MG/Cr, and NAG/Cr, respectively. ATCMR, acute T-cell-mediated rejection; uHSP90, urinary heat shock protein 90; Cr, creatinine; β2MG, β2-microglobulin; NAG, N-acetyl-β-D-glucosaminidase; AUC, area under the curve.

The diagnostic performance of uHSP90/Cr for detecting active tubular injury (ATCMR, CNIN, or BKVN) was evaluated using receiver operating characteristic (ROC) curve analysis (Figure 5). The area under the curve (AUC) was 0.713. At the optimal cutoff value of 0.043 pg/mg Cr, the sensitivity and specificity were 66.7% and 72.7%, respectively, indicating moderate discriminative ability. The diagnostic performance of uHSP90/Cr was also compared with that of other urinary biomarkers, including β2-microglobulin (β2MG)/Cr and N-acetyl-β-D-glucosaminidase (NAG)/Cr (Figure 6, Table 4). The AUC values were 0.713 for uHSP90/Cr, 0.696 for β2MG/Cr, and 0.637 for NAG/Cr. Thus, the diagnostic performance of uHSP90/Cr was comparable to that of β2MG/Cr and superior to that of NAG/Cr.

**Table 4:** Diagnostic performance of creatinine-normalized urinary biomarkers for active tubular injury.

Marker	AUC	Optimal cutoff	Sensitivity (%)	Specificity (%)
uHSP90/Cr, pg/mg creatinine	0.713	0.043	66.7	72.7
β2MG/Cr, μg/mg creatinine	0.696	2.17	74.4	59.7
NAG/Cr, IU/mg creatinine	0.637	0.011	28.2	93.1

Receiver operating characteristic curve analyses were performed to discriminate active tubular injury (acute T-cell-mediated rejection, calcineurin inhibitor nephropathy, or BK virus nephropathy) from all other pathological diagnoses. Optimal cutoff values were determined by maximizing Youden’s index (sensitivity + specificity − 1). The AUC, sensitivity, and specificity are reported at the optimal cutoff values. uHSP90, urinary heat shock protein 90; β2MG, β2-microglobulin; NAG, N-acetyl-β-D-glucosaminidase; AUC, area under the curve.

## Discussion

In this study, we evaluated the clinical significance of uHSP90 in kidney transplant recipients. Several HSPs are released into the urine and have been proposed as biomarkers of renal injury and disease.^8^ Morales-Buenrostro et al reported that uHSP72 is a sensitive early biomarker of acute kidney injury (AKI).^9^ Similarly, Ramires-Sandoval et al. demonstrated that elevated uHSP72 levels were associated with AKI but not with allograft rejection in kidney transplant recipients.^10^ uHSP70 has also been investigated as a biomarker of AKI,^11^ urinary tract infection in children,^12^, ^13^ and obstructive uropathy associated with congenital anomalies of the kidney and urinary tract.^14^ In contrast, the clinical significance of uHSP90 has not previously been investigated, although we previously demonstrated that sHSP90 is a biomarker of acute allograft rejection in kidney transplant recipients.^7^ In the present study, uHSP90 levels were extremely low compared with those of other HSPs, such as HSP70 and HSP72. Nevertheless, we demonstrated that uHSP90/Cr was associated with active tubular injury in kidney allografts and may serve as a non-invasive biomarker reflecting histopathological injury, although it is not specific for allograft rejection. To the best of our knowledge, this is the first study to evaluate uHSP90 levels and their significance with clinical and histopathological findings. The most clinically relevant finding was that uHSP90/Cr levels were significantly elevated in conditions characterized by active tubular injury, including ATCMR, CNIN, and BKVN, whereas no significant increase was observed in BC or IF/TA. These findings suggest that uHSP90 preferentially reflects active tubular injury rather than chronic or relatively mild injury.^15^, ^16^, ^17^, ^18^

Contrary to our initial hypothesis, elevated uHSP90 levels were not specific to ATCMR and therefore do not appear suitable as a non-invasive biomarker capable of replacing allograft biopsy. HSP90 is involved in immune responses, including allograft rejection,^19^ and alloreactive injury targeting vascular endothelial cells may increase circulating sHSP90 levels.^7^ We hypothesized that allorejection-associated immune cell infiltration of tubular epithelial cells would promote the release of HSP90 into the urine. However, the extent of uHSP90 release in tubulitis may be comparable to that observed in renal tubular injury caused by drug toxicity or viral infection. These findings suggest that immune responses within tubular cells may not be the primary determinant of uHSP elevation.

Although the utility of uHSP90 as an ATCMR-specific biomarker appears limited, detectable uHSP90 remained independently associated with active tubular injury after adjustment for sCr and other clinical variables. Notably, the magnitude of this association was greater than that observed for sCr. Furthermore, uHSP90/Cr demonstrated a higher AUC for detecting active tubular injury, compared with conventional urinary biomarkers, such as β2MG and NAG. These findings suggest that uHSP90 is a promising novel biomarker of active tubular injury in kidney allografts. As a urine-based marker, uHSP90 offers the advantage of non-invasive assessment and may complement conventional clinical monitoring. In analyses stratified by biopsy type, uHSP90/Cr was significantly higher in protocol biopsies with active tubular injury despite the absence of a significant difference in sCr. This finding suggests that uHSP90 may complement sCr in identifying histological tubular injury in recipients without clinically apparent graft dysfunction. However, the cross-sectional design does not establish that uHSP90 increases before sCr, and its performance in patients with more severe graft dysfunction requires further investigation. In longitudinal analyses at the individual-patient level, however, changes in uHSP90/Cr were not consistently concordant with changes in the active tubular injury status across repeated biopsies. The temporal relationship between the development or resolution of tubular injury and corresponding changes in uHSP90/Cr remains unclear. A potential time lag between pathological changes and urinary HSP90 levels may partly explain this discordance. Further prospective studies with standardized sampling intervals are required to clarify these temporal relationships and determine the utility of serial uHSP90/Cr measurements for monitoring pathological changes in individual patients. Although uHSP90 cannot replace allograft biopsy, it may help identify ongoing tubular injury and support decisions regarding further diagnostic evaluation. Importantly, uHSP90/Cr levels were significantly associated with Banff scores for interstitial inflammation, tubulitis, interstitial fibrosis, and tubular atrophy, suggesting that uHSP90 reflects histopathological injury at the tissue level. Notably, significant associations were observed with both inflammatory lesions (i and t scores) and chronic lesions (ci and ct scores). However, uHSP90/Cr levels were not elevated in the IF/TA diagnostic category despite the presence of positive ci and ct scores. This apparent discrepancy may be explained by the coexistence of chronic structural lesions and active tubular injury within kidney allografts. Chronic lesions, including IF/TA, frequently develop in allografts with ongoing inflammatory or tubular injury.^15^, ^16^, ^18^ A similar distribution was observed in the present cohort (data not shown), suggesting that the association between uHSP90 and ci/ct scores primarily reflects the coexistence of chronic lesions in cases of active tubular injury, rather than a specific association with chronic injury itself. Furthermore, the lack of a strong correlation between urinary and sHSP90 suggests that uHSP90 is unlikely to be merely a passive filtration product and may instead originate from local processes within the graft. Interestingly, uHSP90 levels were inversely associated with HSP90 immunohistochemical positivity in renal allograft tissue. Previous studies have demonstrated increased HSP90 expression in tubular epithelial cells during the late regeneration phase following ischemic or toxic injury,^20^, ^21^ suggesting that HSP90 contributes to cellular protective mechanisms and tubular regeneration.^8^ Taken together, these findings support the hypothesis that uHSP90 originates from injured and degraded tubular epithelial cells. In contrast, viable tubular cells expressing intracellular HSP90 may not release substantial amounts of HSP90 into the urinary space.

This study has some limitations. First, it was conducted at a limited number of centers, and external validation in independent cohorts is required. Second, although associations with histopathological findings were demonstrated, the biological mechanisms underlying uHSP90 release were not directly investigated. Third, although repeated biopsies were available for a subset of patients, the timing and clinical context of these biopsies were not standardized, and only a limited number of consecutive biopsy pairs showed a change in the active tubular injury status. Therefore, the present data were insufficient to robustly evaluate the longitudinal performance of uHSP90/Cr. Fourth, because multiple biopsy specimens were obtained from some participants, the analyses treated individual biopsies as independent observations and did not account for within-patient correlation. Consequently, the precision of the estimated associations may have been overestimated. Further studies using statistical methods that account for repeated measurements are warranted to confirm these findings.

## Conclusion

uHSP90 levels are elevated in kidney allografts with active tubular injury, including ATCMR, CNIN, and BKVN, although they are not specific to allograft rejection. uHSP90/Cr levels are independently associated with active tubular injury and correlated with histopathological findings, including Banff inflammation and tubular injury scores. In addition, uHSP90 levels are inversely associated with HSP90 expression in renal allograft tissue; this suggests a link between uHSP90 release and tubular epithelial cell injury. These findings indicate that uHSP90 may serve as a clinically useful, non-invasive biomarker of tubular injury in kidney transplant recipients.

## Materials and methods

### Study design and participants

This multicenter prospective clinical study included kidney transplant recipients from Sapporo Medical University, Yamagata University, and Toyama University, Japan.

Kidney transplant recipients who underwent protocol or indication-based allograft biopsy between November 2018 and November 2024 were enrolled. Protocol biopsies were performed as part of routine surveillance irrespective of laboratory evidence of graft dysfunction, whereas indication biopsies were performed in response to clinical or laboratory findings warranting diagnostic evaluation. Urine and blood samples were collected at the time of biopsy. Biopsy specimens with available pathological and clinical data were included in the analysis. The study protocol was approved by the institutional review board (#302–37). Written informed consent was obtained from all participants, and the study was conducted in accordance with the Declaration of Helsinki.

### Sample collection and measurement

Urine and blood samples were collected at the time of biopsy, and supernatants were obtained after centrifugation. uHSP90 concentrations were measured using a human HSP90α enzyme-linked immunosorbent assay (ELISA) kit (ADI-EKS-895; ENZO Life Sciences, Farmingdale, NY, USA) according to the manufacturer’s instructions.

sHSP90 levels were measured using the same assay. To account for variability in urine concentration, uHSP90 levels were normalized to urinary Cr levels and expressed as pg/mg Cr (uHSP90/Cr).

### Additional urinary biomarkers

To compare the diagnostic performance of uHSP90, established urinary biomarkers of tubular injury were also evaluated, including β2MG^22^ and NAG.^23^ These biomarkers were normalized to urinary Cr (β2MG/Cr and NAG/Cr, respectively) and used for comparative analyses.

### Immunohistochemistry

Immunohistochemical staining for HSP90 was performed on renal allograft biopsy specimens for research purposes and was not part of routine clinical assessment. An anti-HSP90 antibody (Abcam, Cambridge, UK; catalog no. ab13495) was used at a dilution of 1:100 following heat-induced antigen retrieval in EDTA buffer at pH 9. For each biopsy, one ×20 field containing abundant tubular epithelium, with the field almost entirely occupied by tissue, was selected by an evaluator who was blinded to the pathological diagnosis and quantitative results. Images were analyzed using Fiji software. Following H-DAB color deconvolution, the DAB and hematoxylin channels were each thresholded from 0 to 200. Hematoxylin-positive pixels were removed from the DAB-positive mask, and the percentage of HSP90-positive area was calculated as the DAB-positive area divided by the total image area. No regions within the selected field were manually excluded. The resulting percentage of HSP90-positive area was used for correlation analyses with urinary HSP90 levels.

### Pathological evaluation

All biopsy specimens were evaluated according to the Banff (2022) classification^24^ by an experienced transplant nephropathologist (Tsuji T.) who was blinded to the urinary and sHSP90 results. Diagnostic categories included acute antibody-mediated rejection (ABMR), ATCMR, BC, chronic active antibody-mediated rejection (CABMR), CNIN, IF/TA, and BKVN. Histopathological lesion scores included interstitial inflammation (i), tubulitis (t), intimal arteritis (v), glomerulitis (g), peritubular capillaritis (ptc), C4d staining, interstitial fibrosis (ci), tubular atrophy (ct), and other Banff parameters. Active tubular injury was defined as the presence of ATCMR, CNIN, or BKVN.

### Statistical analysis

Continuous variables were presented as the median (interquartile range [IQR]) or mean ± standard deviation, as appropriate. Comparisons between two groups were performed using the Mann–Whitney *U* test, and paired comparisons were performed using the Wilcoxon signed-rank test. Comparisons among multiple groups were performed using the Kruskal–Wallis test followed by post-hoc analyses. Categorical variables were compared using Fisher’s exact test. Correlations were evaluated using Spearman’s rank correlation coefficient. ROC curve analysis was performed to assess the diagnostic performance of uHSP90/Cr and other urinary biomarkers for detecting active tubular injury. The AUC, sensitivity, and specificity were calculated. The frequency of active tubular injury was compared between protocol and indication biopsies. Analyses of uHSP90/Cr, detectable urinary HSP90, and sCr were additionally stratified by biopsy type. For patients with multiple biopsies, consecutive biopsy pairs in which the active tubular injury status changed were identified. Changes in uHSP90/Cr were descriptively classified as concordant, discordant, or unchanged according to the direction of change relative to the active tubular injury status; no inferential statistical testing was performed for this analysis. Multivariable logistic regression analysis was conducted to identify factors independently associated with active tubular injury. Age at biopsy, time from transplantation to biopsy, sCr, and detectable urinary HSP90 (defined as uHSP90 > 0 ng/mL) were selected based on their clinical relevance as potential correlates or confounders of active tubular injury. Biopsy type (protocol or indication biopsy) was also included in the multivariable model. ORs and 95% CIs were calculated. A two-sided P value < .05 was considered statistically significant. All statistical analyses were performed using JMP Student Edition 19 (SAS Institute Inc., Cary, NC, USA).

## Funding and support

This work was supported by JSPS KAKENHI (Grant Numbers 23K15437 to Maehana T and 24K11808 to Tanaka T). The funder had no role in the study design, data collection, data analysis, data interpretation, manuscript preparation, or the decision to submit the article for publication.

## CRediT authorship contribution statement

**Kimihito Tachikawa:** Writing – original draft, Visualization, Investigation, Formal analysis, Data curation, Conceptualization. **Toshiaki Tanaka:** Writing – review & editing, Supervision, Methodology, Conceptualization. **Takeshi Maehana:** Writing – review & editing, Conceptualization. **Tetsuya Shindo:** Writing – review & editing. **Yuki Kyoda:** Writing – review & editing. **Kohei Hashimoto:** Writing – review & editing. **Hayato Nishida:** Writing – review & editing, Resources. **Hiroshi Kitamura:** Writing – review & editing, Resources. **Takahiro Tsuji:** Writing – review & editing. **Naoya Masumori:** Writing – review & editing, Supervision, Project administration.

## Declaration of Generative AI and AI-Assisted Technologies in the Writing Process

During the preparation of this manuscript, the authors used ChatGPT (OpenAI) to assist with English language editing and improve readability. The authors reviewed and edited the content as necessary and take full responsibility for the content of the publication.

## Declarations of interest

The authors declare that they have no known competing financial interests or personal relationships that could have appeared to influence the work reported in this study.

## Data Availability

The authors do not have permission to share data.
