# Supplementary material for: Urinary HSP90 as a non-invasive biomarker of active tubular injury in kidney transplant recipients: A multicenter prospective study
Source: Cell Stress Chaperones. 2026 Aug 26;31(6):100209. doi: 10.1016/j.cstres.2026.100209 (PMC13572301; doi:10.1016/j.cstres.2026.100209)
Supplement: Supplementary file 1 [file mmc1.docx]

| **Supplementary Table 1. Clinical Parameters According to Active Tubular Injury Status, Stratified by Biopsy Type** | | | | | | |
| --- | --- | --- | --- | --- | --- | --- |
| Variable | Protocol biopsy:  No active tubular injury (n=179) | Protocol biopsy:  Active tubular injury (n=21) | P value | Indication biopsy:  No active tubular injury (n=37) | Indication biopsy:  Active tubular injury  (n=18) | P value |
| uHSP90/Cr,  pg/mg creatinine | 0 [0–0.116] | 0.519 [0–1.008] | <0.001 | 0 [0–0.078] | 0.299 [0–1.481] | 0.004 |
| Detectable uHSP90,  n (%) | 52 (29.1) | 14 (66.7) | 0.001 | 9 (24.3) | 12 (66.7) | 0.004 |
| Serum creatinine,  mg/dL | 1.30 [1.04–1.51] | 1.38 [1.02–1.73] | 0.486 | 1.70 [1.46–2.33] | 2.69 [1.75–4.42] | 0.017 |
| Data are presented as median [interquartile range] or number (percentage). P values were calculated using the Mann–Whitney U test for continuous variables and Fisher’s exact test for detectable uHSP90. Serum creatinine data were unavailable for two indication biopsies with active tubular injury; therefore, the serum creatinine analysis in this group included 16 biopsies. uHSP90, urinary heat shock protein 90; Cr, creatinine | | | | | | |
